# Use of nares swab to de-escalate vancomycin for patients with suspected methicillin-resistant *Staphylococcus aureus*

**DOI:** 10.1017/ash.2023.444

**Published:** 2023-10-09

**Authors:** Joshua Gentges, Nadeem El-Kouri, Tashrique Rahman, Nasir Mushtaq, Ed Hudson, David Scheck

**Affiliations:** 1 Department of Emergency Medicine, The University of Oklahoma School of Community Medicine (OUSCM), Tulsa, OK, USA; 2 Providence Hospital, Gresham, OR, USA; 3 Public Health Epidemiology, Tulsa, OK, USA; 4 Hillcrest Hospital, Tulsa, OK, USA; 5 Infectious Disease/Hospital Epidemiologist Hillcrest Medical Center, Tulsa, OK, USA

## Abstract

**Introduction:**

According to the US Center for Disease Control and Prevention, 30%–50% of antibiotic use in hospitals is unnecessary or inappropriate. The coronavirus disease 2019 pandemic further complicates antibiotic use leading to greater initiation of empiric antibiotics. The result is antibiotic overuse and increased duration of unnecessary therapy. Vancomycin is a drug of last resort, primarily relegated to the treatment of Methicillin-Resistant *Staphylococcus aureus* (MRSA). De-escalating vancomycin can mean waiting on MRSA culture results, which may take up to 96 h. Nares screening for MRSA is shown to possess high negative predictive value for ruling out suspected MRSA pneumonia, intra-abdominal infections, and bacteremia.

**Methods:**

This before-and-after study examines the impact of vancomycin therapy de-escalation due to absence of MRSA colonization detected via PCR assay of nares swabs. An intervention with providers using SMART goals was designed to increase nasal swabbing for MRSA and ultimately decrease vancomycin use at a large, tertiary-care urban hospital.

**Results:**

There was a significant increase in use of vancomycin nares swabs (28/150 vs 48/100, *p* = 0.040) in the immediate pre/postintervention period, and significant decreases in vancomycin usage days/1,000 patient days of 2.34% per month (*p* = 0.039) over a two year period after the intervention.

**Conclusion:**

An intervention using PCR nares swabs to detect MRSA led to significant, lasting decreases in vancomycin usage at this hospital. Similar interventions should be planned at hospitals experiencing overuse of this antibiotic.

## Introduction

The inception of the coronavirus disease 2019 (COVID-19) virus (severe acute respiratory coronavirus virus 2 [SARS-CoV-2]) has reversed decades of progress in antimicrobial stewardship.^1^ Uncertainty regarding this disease prompted healthcare providers to overtreat patients with empiric antibiotics. A retrospective study of 716 hospitals conducted on adult and pediatric inpatient hospitalizations at US hospitals during March–October 2020 showed that 77.3% of inpatients diagnosed with COVID-19 received at least 1 antibiotic per day during their stay, and 81.3% of those who received an antibiotic were started on admission.^2^ Patients with COVID-19 infrequently have bacterial coinfections. A multi-center study showed 9.5% of the enrolled 905 COVID-19 patients had a clinically diagnosed bacterial coinfection.^3^ A meta-analysis showed that 7% of hospitalized COVID-19 patients had a bacterial coinfection (*N* = 2183, 95% CI 3%–12%).^4^

The overuse of empiric antibiotics instigated a domino sequence of events; most important of which is the re-emergence of antibiotic-resistant infections. Resistant hospital infections and deaths increased at least 15% during the first year of the pandemic.^5^ Methicillin-resistant *Staphylococcus aureus* (MRSA) infections have increased by 13% during the pandemic. Infection with MRSA has its own set of challenges and is associated with extended length of hospital stay (6–14 d), inflated hospital costs ($3220–$9606), and a hospital mortality rate of 0%–3.58%.^6^ Vancomycin, as an anti-MRSA agent, is often prescribed and continued as empiric therapy.^7^

A core tenet of antibiotic stewardship entails continued re-evaluation of the needs for therapy and appropriate de-escalation. The decision to de-escalate vancomycin can be mitigated with negative MRSA culture, which may take up to 96 h.^8^ In the meantime, patients can be needlessly at risk for adverse events such as preventable allergic reactions (i.e. vancomycin flushing syndrome, eosinophilia, systemic symptoms syndrome, etc.),^9^ vancomycin-resistant infections (*Staphylococcus aureus*, *Enterococcus* strains),^10,11^ kidney injuries,^12^ and drug-drug interactions.^13^ Vancomycin requires critical drug level monitoring, frequent lab draws, and an expert understanding of pharmacokinetic concepts (i.e. area under the curve, minimum inhibitory concentration).^14^ Earlier de-escalation can reduce total healthcare expenses^15^ (around $108 per patient encounter), which frees resources for other hospital missions.

Nasal colonization with MRSA has a high NPV (>94%) for lower respiratory tract infection.^16–19^ Polymerase chain reaction (PCR) of nasal swabs has a 98% NPV for MRSA colonization^20^ and can yield results in as little as 2 h. Nasal swab MRSA PCR testing has been shown to reduce the duration of empirical MRSA-targeted therapy by approximately 2 d (*p* < 0.001) without worsening clinical course and lowers the incidence of acute kidney injury (*p* = 0.02).^21^ A retrospective review of all consecutive patients from April 2010 to 2015 at McGill University Health Centre, an 832-bed hospital, suggested that MRSA screening could help avoid empiric vancomycin therapy and its complications in stable patients and settings with low-to-moderate proportions (20%–40%) of MRSA bacteremia.^22^ Hennessy et al.^23^ found a positive nasal screen to be an independent indicator for suspected MRSA intra-abdominal infections; a negative screen can rule out the possibility of an MRSA infection. These findings warrant the need to incorporate nasal screenings as a clinical tool in the setting of a suspected MRSA infection.

## Research design and methods

This single-center study of a quality improvement initiative examines vancomycin overuse between May 2020 and June 2021, before and after implementation of this quality improvement initiative. The site is a large, inner-city tertiary referral center with 48,000 ED visits a year and is a burn center. Inclusion criteria include patients at least 18 years of age, who are suspected of having potential for MRSA infections. Suspected infections included pneumonia, intra-abdominal infections, bacteremia, and skin infections. Urinary tract infections were excluded. Patients undergoing this intervention were swabbed in the nares prior to administration of vancomycin therapy. Bilateral nares were swabbed by protocol and Cepheid Xpert MRSA assay was performed in the GeneXpert® System (Xpert MRSA). The primary outcome is if provider intervention increases nares MRSA PCR screening. The secondary outcome is if vancomycin usage declines after provider intervention.

We measured vancomycin therapy days before and after the intervention along with the number of swabs collected after implementation of the intervention. Policy was implemented at the hospital level by dissemination of the initiative through the Pharmacy and Therapeutics Committee and the Antibiotic Stewardship Council. The initiative required buy-in from providers. To this end, pharmacists provided education to prescribers about the significance of nasal screening as a clinical tool to de-escalate vancomycin therapy in October 2020. From November 2020, prescribers were guided to order nasal screening when MRSA infection was suspected. The multidisciplinary team consisting of physicians, mid-level providers, and pharmacists was encouraged to utilize the negative results of the nares swab to de-escalate vancomycin therapy. Providers retained freedom to 1) decline to order nasal swabbing and 2) discount swab results and treat patients per usual care. We collected no individual patient data.

The intervention strategy was designed with SMART goals in mind. These goals are listed below:

Specific – Nasal swab for MRSA in pneumonia/suspected bacteremia

Measurable – Measure number of swabs and number of vancomycin therapy days/pt.

Achievable – Policy change through P&T, Antibiotic Stewardship Committee

Realistic – Behavior change requires buy-in from providers

Timely – Expect intervention to take 6–12 mo for changes to move the needle much.

## Statistical methods

The design was a pre/post quality improvement study. We reviewed vancomycin orders completed in the study time period to analyze practice patterns of nasal MRSA PCR and correlate that with vancomycin therapy duration and exposure to therapy. This was done for 3 mo preintervention and 2 mo postintervention, allowing 2 mo around the time of the intervention to educate providers. We measured intervention effectiveness during the study period and adapted as needed. Change in vancomycin utilization pre and postintervention was examined by joinpoint regression analysis. Weighted least squares analysis was performed with the options of homoscedasticity and estimation of first-order autocorrelation from the data. Average monthly percent change and 95% confidence interval (95%CI) along with *p*-value were calculated to determine significant trends in vancomycin utilization estimates during the pre and postintervention periods. Interrupted time series analysis was also conducted. Autoregressive error model with stepwise autoregressive process was performed to obtain maximum likelihood parameters. The null hypothesis is that no difference exists between vancomycin utilization rates pre/postintervention.

## Results

**Figure 1.**
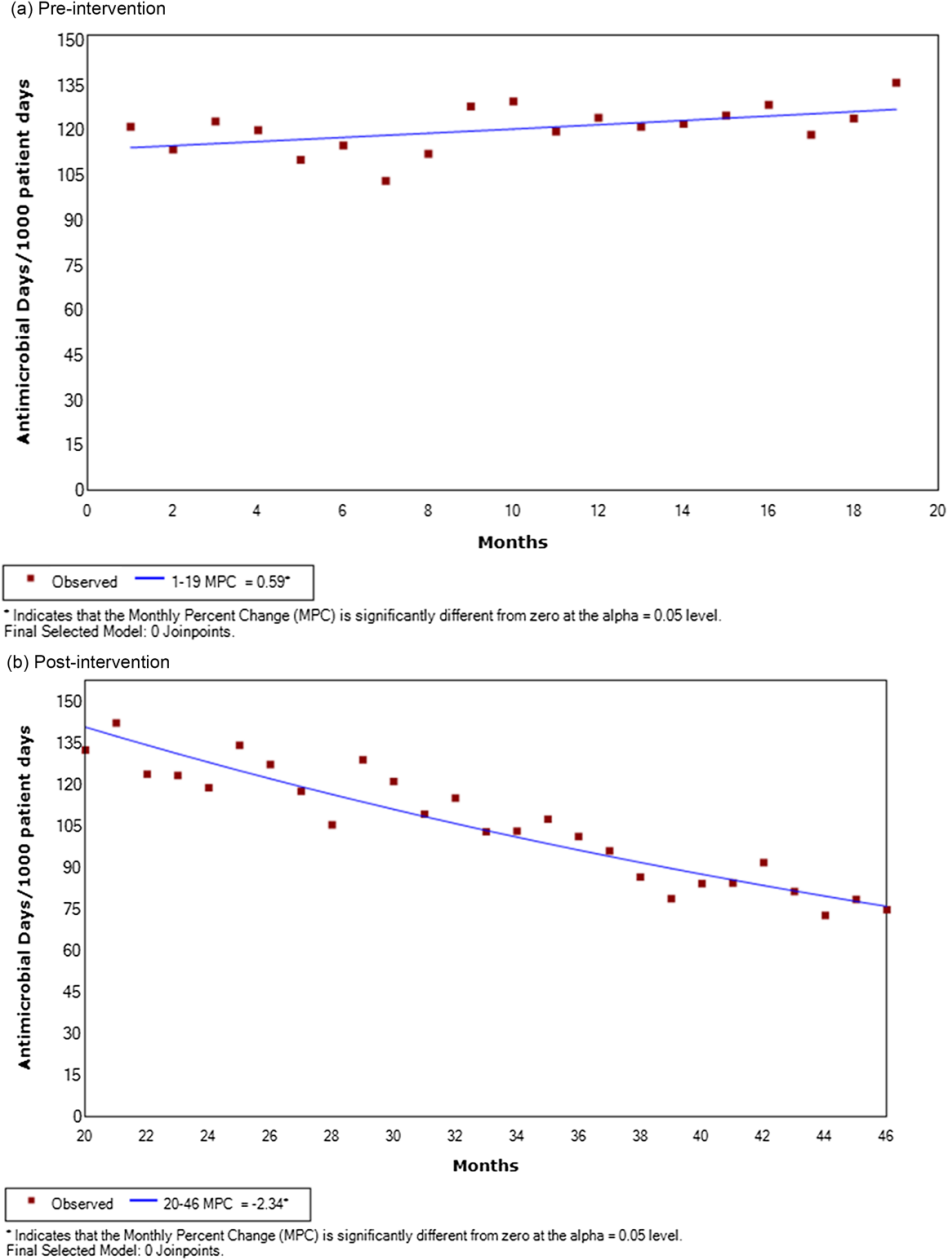
Trends in vancomycin utilization rates pre and postintervention. (a) Preintervention. (b) Postintervention.

A random chart audit of 50 MRSA-positive patients per month compared 20 mo of vancomycin use before the intervention in August through October 2020 to use during and after the intervention until data collection ended in November 2022. We measured the use of nasal swabs to test for MRSA (the “S” in our SMART goals) directly after the intervention by a random chart audit for the intervention months and the immediate postintervention months of November and December 2020. These increased 28/150 (18.7%) to 48/100 (48%), a statistically significant (*p* = 0.04) increase, showing that our process goal was met. As the intervention continued, there were large changes in vancomycin utilization at the hospital (Figure 1).

Durbin–Watson test indicated positive correlation (Durbin–Watson D statistic = 0.475, *p* < 0.001) between observations (i.e., vancomycin utilization) at different time points. Results of the joinpoint regression analysis showed that for the preintervention period, average monthly percent change in vancomycin utilization significantly increased by 0.59% (95%CI: 0.12, 1.06, *p* < 0.016), whereas, during the postintervention period, it significantly decreased by an average monthly change of −2.34% (95%CI: −2.64, −2.04%, *p* < 0.001). Results of interrupted time series analysis indicated significant impact of intervention on vancomycin utilization (*β* = 16.79, SE = 7.89, *p* = 0.0394).

## Discussion

Antibiotic resistance is a wicked problem, in the classical public health sense of the term.^24^ As such, we lean on systems sciences thinking to address this multifactorial issue. Our educational intervention is aimed at providers and pharmacists and is meant to empower both groups to work together to reduce unnecessary vancomycin therapy. Early collection of the nasal swabs to determine MRSA colonization mitigates decision-making downstream and provides a clear cause for de-escalation of therapy.

Our study demonstrates a viable de-escalation strategy for vancomycin. Implementation of multi-level education between pharmacists and providers alongside purchase of MRSA PCR technology allows for risk stratification of MRSA disease based on MRSA colonization. The early discontinuation of vancomycin decreased days of therapy. We did not perform a cost analysis, but a 2017 study^15^ showed cost savings from MRSA swabbing of 108 dollars per patient encounter. Notably, MRSA screening to guide vancomycin therapy should be avoided in patients with recent nasal decolonization before screening and MRSA infection within 30 d before admission.^25^ In patients with structural lung disease (such as cystic fibrosis or bronchiectasis), MRSA nares screens may be discordant because colonization occurs more frequently in the lower respiratory tract and should be avoided. Moreover, in critically ill intensive care unit patients, more cautious de-escalation may be considered with de-escalation at 48 h because 98% of positive blood cultures for Staphylococcus aureus occur within this time.

Antibiotics are still of great utility and represent a cornerstone therapy in many high-mortality clinical contexts, as evidenced by the Surviving Sepsis Campaign.^26^ Ferrer et al., for example, demonstrated a proportional increase in mortality risk for each hour delay in administration of antibiotics in their multinational retrospective review of sepsis management in nearly 18,000 patients.^27^ The intent, therefore, is not to eliminate their use altogether or even lessen the use of empiric application, but rather to minimize patients’ exposure when unnecessary, as well as the associated unnecessary adverse effects, increased costs, and misallocation of resources.

In 2009, 11% of the overall US healthcare expenditures were attributable to medications,^28^ equaling between $249.9 and 300 billion.^29^ Antibiotics comprised one of the most costly categories with an estimated $10.7 billion cumulative expenditure, surpassed only by antineoplastic and hemostatic modifiers (antibiotic expenditures by medication, class, and healthcare setting in the United States, 2010–2015). The previous studies, however, are likely a significant underestimation of the overall impact as they do not account for downstream effects including adverse events, prolonged healthcare encounters, or subsequent related healthcare encounters.

To our knowledge, this is among few studies of an intervention to target antibiotic stewardship at the level of a medication class. Previous programs at levels ranging from local to national have focused on prespecified age groups and conditions, such as acute otitis media or bronchitis.^30^ Vancomycin is susceptible to unique de-escalation given its relatively narrow therapeutic intent of targeting MRSA and the highly correlative nasal PCR testing available.

The United Nations says that antimicrobial resistance could lead to 10 million deaths by 2050.^31^ Antimicrobial resistance could be the primary cause of death in the upcoming years and could lead to an economic crisis worse than that of 2008.^32^ A common yet successful measure to prevent antibiotic resistance in a hospital setting is antibiotic de-escalation. It is imperative that healthcare professionals continue to explore de-escalation strategies before the burden of antimicrobial resistance cripples our healthcare system.

## Limitations

This was a single-center study, and results may not be applicable to other hospitals; differences in culture, available laboratory tests, patient population, and personnel available for the intervention could vary the results at other sites. Changes in providers and seasonal effects could also confound the results, but this issue is mitigated somewhat by the extended (almost 4 yr) reporting time of sustained decreases in vancomycin use. This strategy is unique to the use of vancomycin because of the availability of MRSA nasal swabbing and cannot be extrapolated to other antibiotics. We did not control for patient illness severity or individual patient factors since the intervention and outcomes were population-level findings. There were no adverse outcomes (such as untreated MRSA) reported during the study period to the Antibiotic Stewardship Council at our site, but this study was not designed to assess such outcomes. Although we did not identify adverse outcomes, further study showing non-inferiority of our intervention related to patient morbidity and mortality is warranted.

## Conclusion

Nasal swabbing for MRSA increased significantly and vancomycin use/days decreased significantly over an extended period after implementing a hospital-wide intervention designed to increase the use and knowledge of MRSA nasal swabbing. Other centers with overuse of this antibiotic should design local interventions using SMART goals similar to ours. Further areas of study should include patient-level outcomes and demographic data associated with this type of intervention.
